# A proteome map of the zebrafish (*Danio rerio*) lens reveals similarities between zebrafish and mammalian crystallin expression

**Published:** 2008-04-25

**Authors:** Mason Posner, Molly Hawke, Carrie LaCava, Courtney J. Prince, Nicholas R. Bellanco, Rebecca W. Corbin

**Affiliations:** 1 Department of Biology, Ashland University, Ashland, OH; 2 Department of Chemistry, Ashland University, Ashland, OH

## Abstract

**Purpose:**

To characterize the crystallin content of the zebrafish lens using two-dimensional gel electrophoresis (2-DE). These data will facilitate future investigations of vertebrate lens development, function, and disease.

**Methods:**

Adult zebrafish lens proteins were separated by 2-DE, and the resulting spots were identified by matrix-assisted laser desorption/ionization time of flight mass spectrometry (MALDI-TOF MS). The relative proportion of each crystallin was quantified by image analysis, and phosphospecific staining was used to identify phosphorylated α-crystallins. The proportion of each crystallin in the soluble and insoluble fraction of the lens was also determined by resolving these lens fractions separately by 2-DE.

**Results:**

α-, β-, and γ-crystallins comprised 7.8, 36.0, and 47.2% of the zebrafish lens, respectively. While the α-crystallin content of the zebrafish lens is less than the amounts found in the human lens, the ratio of αA:αB crystallin is very similar. The phosphorylation pattern of zebrafish αA-crystallins was also similar to that of humans. The most abundant γ-crystallins were the diverse γMs, comprising 30.5% of the lens. Intact zebrafish crystallins were generally more common in the soluble fraction with truncated versions more common in the insoluble fraction.

**Conclusions:**

While the total α- and γ-crystallin content of the zebrafish lens differs from that of humans, similarities in α-crystallin ratios and modifications and a link between crystallin truncation and insolubility suggest that the zebrafish is a suitable model for the vertebrate lens. The proteome map provided here will be of value to future studies of lens development, function, and disease.

## Introduction

Fishes have become a valuable tool for the comparative study of vertebrate eye diseases [1]. In particular, the zebrafish has been used to investigate eye development [2-7], glaucoma [8,9], retinal degeneration and regeneration [10,11], and cataract [12]. This species’ short generation time and nearly completed genome facilitate its use as a model vertebrate. Furthermore, the genes for a large number of lens crystallins, the major structural and protective proteins of the lens, have been cloned from zebrafish [13-16]. While several studies have examined the expression of zebrafish crystallins at the mRNA level [3,12,14-16], there are few data on the relative proportions or posttranslational modifications of the proteins themselves. Previous attempts to calculate the relative proportions of each crystallin family have been hampered by the inability to separate α- and β-crystallins by size exclusion chromatography [14,17,18]. A proteomics approach using two-dimensional electrophoresis (2-DE) such as the one used in this study will increase the utility of zebrafish as a model for eye disease and provide valuable data for future investigations of lens development.

2-DE has been used successfully to describe the crystallin content and modifications that occur in the mammalian [19,20] and chicken [21] lens. Protein truncation and other modifications such as phosphorylation, deamidation, and methylation alter the protective chaperone activity of α-crystallins and the solubility of β- and γ-crystallins [20,22-24]. These posttranslational modifications accumulate with age and increase the likelihood of cataract. We previously performed a 2-DE analysis of zebrafish lens α-crystallins [16], but very few other studies have used the technique on zebrafish tissues [25,26]. A more detailed proteome map of the zebrafish lens will facilitate the study of age and stress-related changes to crystallins and their impact on lens function. Comparison of these data with those from other vertebrate species can provide additional insight into lens function and disease.

In this study, we used 2-DE and mass spectrometry (MS) to detail the expression of crystallins in the zebrafish lens. Although we found that α-crystallin levels are lower than in the mammalian lens, the ratio between αA- and αB-crystallins is very similar, suggesting that these proteins play similar roles in both groups. We also show that similar to the mammalian lens, zebrafish αA-crystallin contains many phosphorylated isoforms and that truncations of zebrafish crystallins are correlated with protein insolubility. These data support the use of the zebrafish as a model for studying the development, function, and aging of the vertebrate lens.

## Methods

### Separation and visualization of lens proteins

Six to eight lenses were collected from adult wild type zebrafish and homogenized in 600 µl of sample buffer (8 M urea, 2% CHAPS, 50 mM DTT, 0.2% Bio-Lyte 3/10 ampholyte, and 0.001% bromophenol blue; Bio-Rad, Hercules, CA) to solubilize total lens protein. Fish were obtained from aquarium stores, and the exact ages were not known. All fish were euthanized using methods authorized by Ashland University’s Institutional Animal Care and Use Committee. Lens homogenates were centrifuged at 15,000x g for 20 min to remove any unsolubilized material, and the protein in the supernatant was quantified using the RC DC protein assay kit (Bio-Rad). One hundred and fifty micrograms of lens homogenate were focused on immobilized pH gradient (IPG) strips (11 cm; pH 3–10 nonlinear, pH 5–8, and pH 4–7; Bio-Rad). Second dimension separation was performed on 12% SDS–PAGE gels with subsequent Coomassie staining. Gels were digitally imaged with a Kodak 440 CF Imagestation (Kodak, Rochester, NY), and the proportion of total protein found in each spot on the pH 3–10 nonlinear gels was quantified by densitometry using Kodak 1D Image Analysis software (Kodak). The proportion of each crystallin was calculated as the mean of three separate lens samples. Some gels were stained with Pro-Q Diamond phosphoprotein gel stain (Invitrogen, Carlsbad, CA) to identify phosphorylated proteins. These gels were subsequently stained with SYPRO Ruby protein gel stain to visualize all protein spots (Invitrogen).

**Figure 1:**
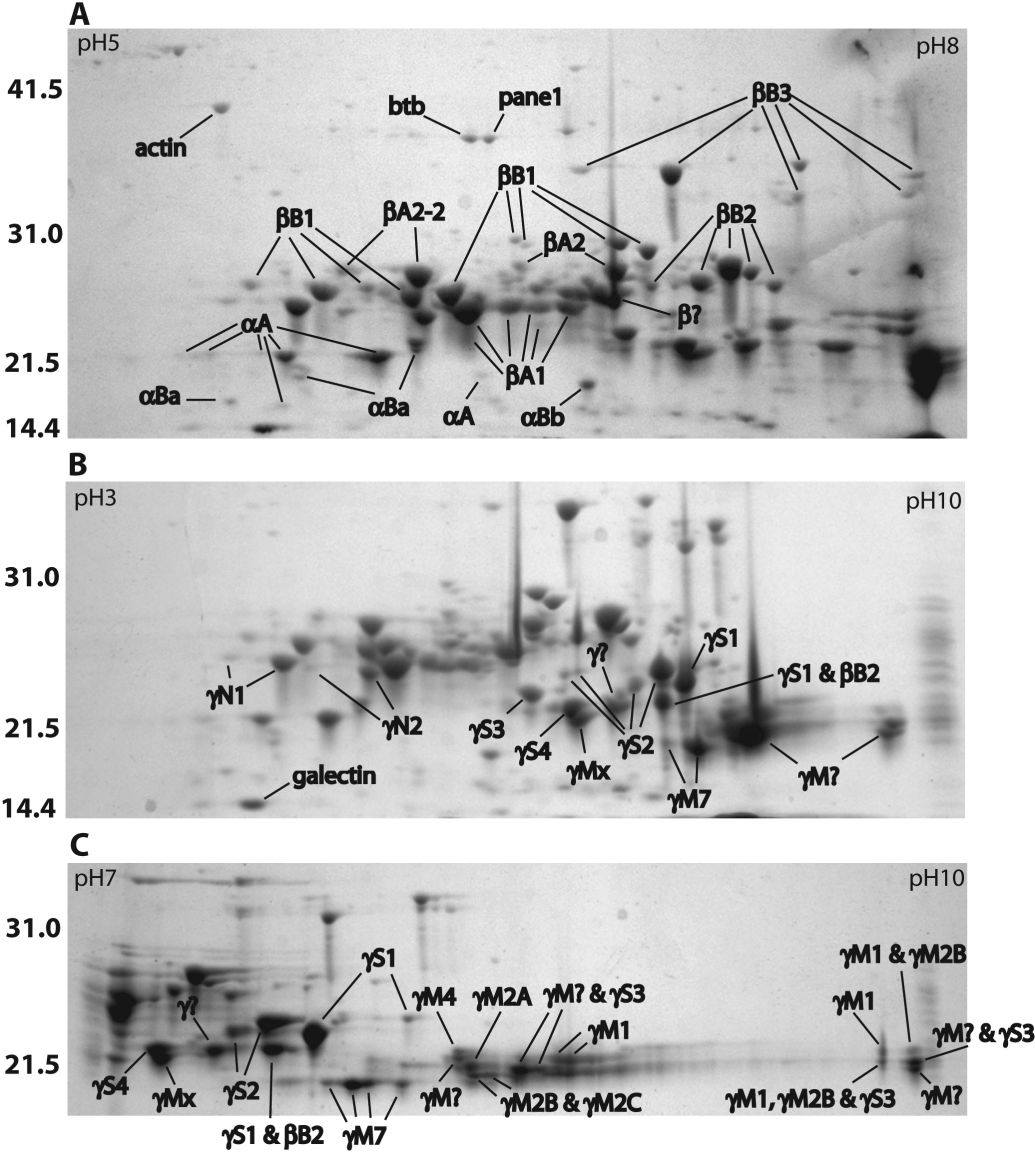
Two-dimensional electrophoresis (2-DE) profiles of total adult zebrafish lens protein. Separation was performed on 11 cm IPG strips with pH gradients of 5–8 (**A**), 3–10 nonlinear (**B**) and 7–10 (**C**). Gels were Coomassie stained and spots were identified by MALDI-TOF MS. Mass standards are indicated on the left in kiloDaltons. Spots labeled with a “?” contain two or more crystallins from the indicated family. Accession numbers for each crystallin are shown in Table 1. The accession numbers for four identified non-crystallins are as follows: actin (AAO38846), BTB (POZ) domain containing protein 2 (NP_001038557), proliferation associated nuclear panel 1 (Pane1; NP_991153), and galectin-related inter-fiber protein (XP_684452).

### Identification of two-dimensional gel electrophoresis spots by matrix-assisted laser desorption/ionization time of flight mass spectrometry

Gel spots were selected for peptide mass fingerprinting. Coomassie stained spots were excised from SDS–PAGE gels, destained, and digested overnight with proteomics grade trypsin (Sigma, St. Louis, MO). Digest solutions were acidified with 0.1% trifluoroacetic acid and mixed with the matrix alpha-cyano-4-hydroxycinnamic acid. Samples were analyzed by matrix-assisted laser desorption/ionization time of flight (MALDI-TOF) mass spectrometry using a Bruker MicroFLEX instrument (Bruker Daltonics, Billerica, MA). Externally calibrated, positive-ion mass spectra were obtained in reflection mode. The resulting mass spectra were compared to theoretical peptide mass maps using ProteinProspector software and the NCBI non-redundant protein database. Further confirmation of protein identity was performed on selected samples by the analysis of post-source decay fragments of tryptic peptide ions.

### Two-dimensional gel electrophoresis analysis of soluble and insoluble protein fractions

Zebrafish lenses from 12 individuals were homogenized with a Wheaton homogenizer (Wheaton Science Products, Millville, NJ) in 800 µl of 20 mM sodium phosphate buffer containing protease and phosphatase inhibitors (Roche Molecular Biochemicals, Basel, Switzerland). The resulting homogenate was centrifuged at 15,000x g for 15 min. The supernatant containing the soluble protein fraction was removed, and the insoluble pellet was resuspended in 800 µl of 2-DE sample buffer (see above; Bio-Rad). The protein content of the soluble and insoluble fractions was quantified using a Bradford assay and RC DC protein assay, respectively (Bio-Rad). Both samples were diluted with 2-DE sample buffer to apply 150 μg to 11-cm IPG strips (pH 5–8) before isoelectric focusing.

**Figure 2:**
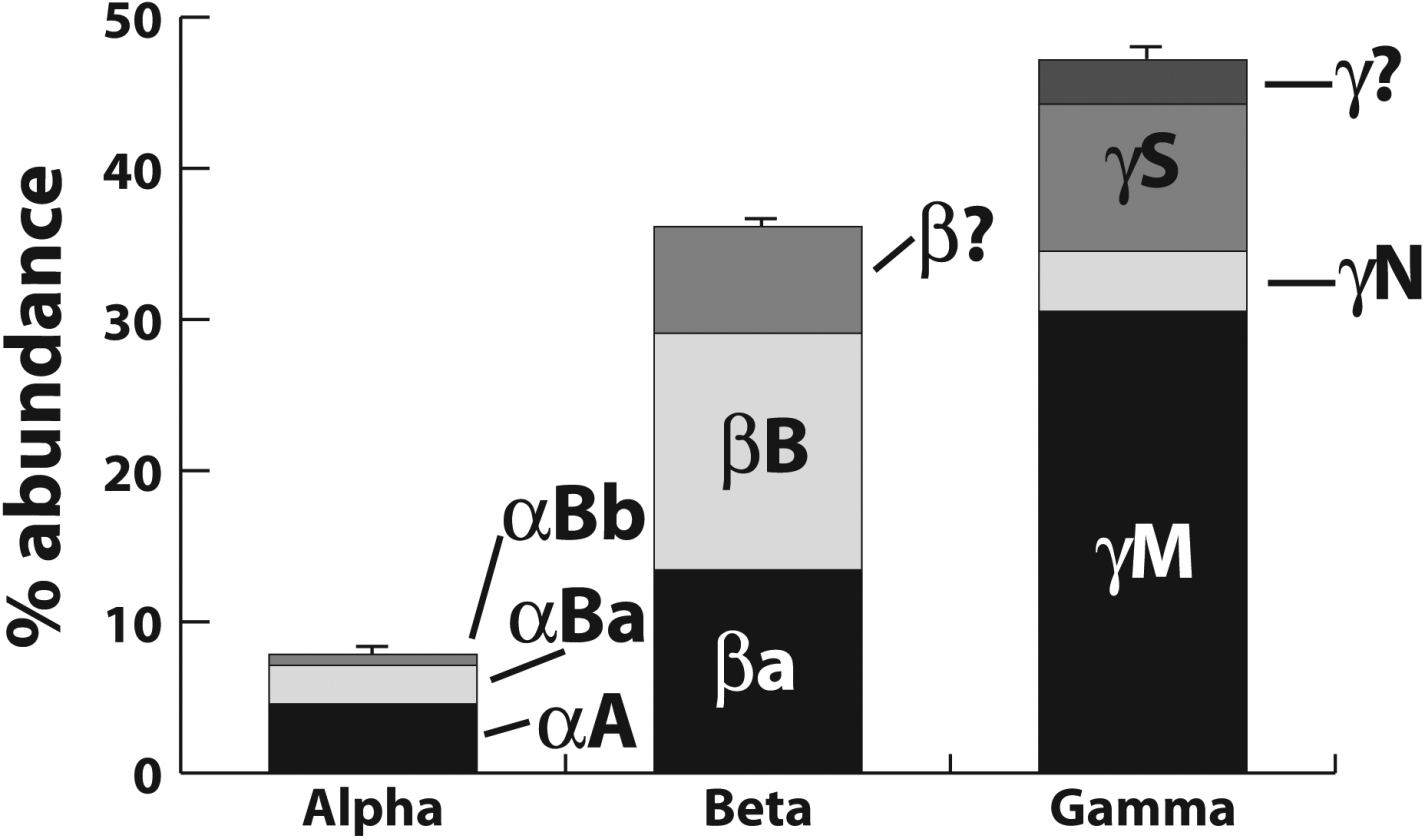
Proportion of α-, β-, and γ-crystallins in the zebrafish lens determined by densitometry of Coomassie stained two-dimensional electrophoresis gels. Each crystallin family is further subdivided as labeled. Error bars show standard deviation for three separate lens samples. Abundance of protein spots that were identified only to family or contained more than one member of each family are indicated with question marks.

## Results

Our 2-DE analysis resolved over 80 protein spots. Sixty of these spots were identified by MALDI-TOF MS as one of 28 different crystallins, and another four were identified as non-crystallins (Figure 1). The large number of crystallin spots reflects an abundance of posttranslational modifications. Similar to data from a previous study [17], we found that the γ-crystallins were the most abundant crystallin family in the fish lens (Figure 2). However, our data are the first to show that of the γ-crystallins, the aquatic specific γMs were the most abundant (Figure 2). Furthermore, the 7.8% total α-crystallin shown here in the zebrafish lens is lower than previously reported values for fish lenses [27,28]. Details for each crystallin family are given in the following sections.

### Quantitation and modification of α-crystallins

Few studies have calculated the proportion of each major crystallin family in the fish lens. Furthermore, since it was recently shown that the zebrafish lens contains a second αB-crystallin [16], no study has determined the relative amounts of all three zebrafish α-crystallins. We found α-crystallins in 13 different 2-DE gel spots, suggesting several posttranslational modifications (Figure 1A). Total α-crystallin content in the lens was 7.8% with a 6.4:3.6:1 ratio of αA:αBa:αBb (Figure 2, Table 1). This value is very similar to the 3:2 ratio of αA:αB crystallin reported for the adult human lens [29].

**Table 1:** Zebrafish lens crystallins identified by MALDI-TOF MS from two-dimensional electrophoresis gels.

**Crystallin**	**Percent abundance**	**Number of spots**	**Protein accession number**	**Zebrafish chromosome**	**Human chromosome**
αA	4.57±0.22	8	AAH83177	1	21q22.3
αBa	2.56±0.25	4	AAD49096	15	11q22.3-q23.1
αBb	0.72±0.04	1	NP_001002670	5	h
βA1	8.02±0.82*	6	NP_001002410	15	17q11.2
βA1–2	nq	3	AAY18967	14	h
βA2	2.63±0.18	2	AAY18965	?	2q34-q36
βA2–2	2.79±0.64*	3	AAZ66113	?	h
βA4	nq	2	AAY18966	?	22q11.2-q13.1
βB1	7.58±1.20*	11	AAH76186	10	22q12.1
βB2	4.86±0.60*	9	AAY18969	8	22q11.23
βB3	3.21±1.44	6	AAY18970	5	22q11.23
γM1	nq	11	NP_001007786	2	na
γM2a	nq	4	NP_001018131	2	na
γM2b	nq	10	NP_001018619	2	na
γM2c	nq	9	NP_001007784	2	na
γM2d1	nq	1	AAH95033	9	na
γM2d2	nq	1	NP_001038331	?	na
γM3	nq	8	NP_001007787	8	na
γM4	nq	3	NP_001007792	21	na
γM6	nq	1	NP_001018630	2	na
γM7	4.68±0.31	5	NP_001018631	2	na
γMx	1.14±0.33*	2	NP_001013280	12	na
γN1	2.23±0.27	2	NP_001007785	2	7q36.1
γN2	1.75±0.48	3	NP_001003428	24	h
γS1	2.16±0.55*	4	NP_001013294	22	3q25-qter
γS2	5.09±1.21	4	XP_696410	9	h
γS3	1.22±0.15*	7	AAH93207	9	h
γS4	1.26±0.30*	19	NP_001013293	9	h

Percent abundance of each protein was calculated by densitometry of three Coomassie stained gels. Error is standard deviation (n=3). Percent abundance for some proteins was not quantifiable (nq) because they only occurred in mixed spots with other proteins. Asterisks indicate crystallins that were found in both individual spots and mixed spots. Therefore, these percent abundances are underestimates of the true protein abundance. The number of spots containing each crystallin is also indicated. The presence of more than one spot per crystallin is due to truncations or other modifications. Protein accession numbers are for GenBank. Chromosomal location for the corresponding zebrafish and human crystallin genes were identified using zfin and GenBank, respectively. A “?” indicates an unknown chromosomal location; “na” indicates the lack of a human homolog for a specific zebrafish crystallin; and “h” indicates that only one human homolog occurs for multiple paralogous zebrafish genes.

**Figure 3:**
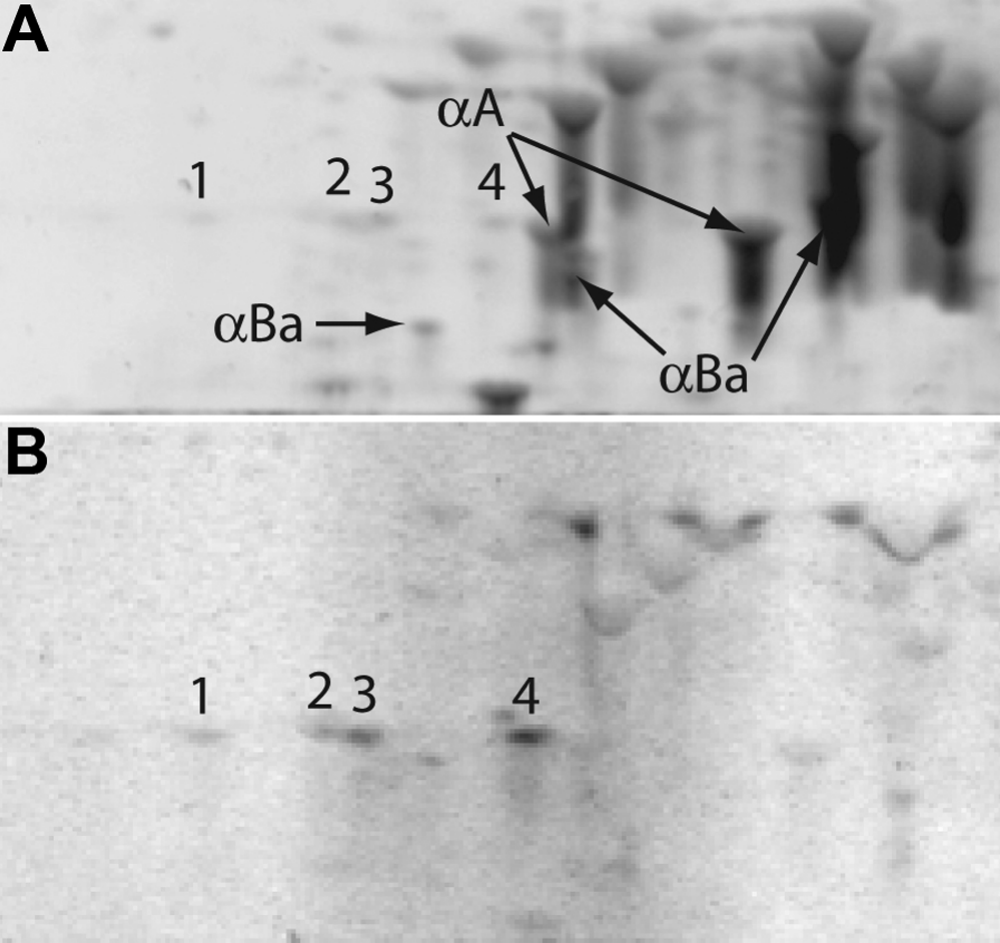
Phosphoprotein staining of two-dimensional electrophoresis gels (pH 5–8) indicates phosphorylated αA-crystallins. Numbers identify the equivalent αA-crystallin spots on gels stained with the total protein stain (**A**) and the phosphoprotein-specific stain (**B**). Labels and arrows indicate α-crystallin spots that were not detected by the phosphoprotein stain.

Phosphorylation has been shown to modify the function of α-crystallins [30-32]. We used a phosphoprotein-specific stain to identify phosphorylated α-crystallin spots (Figure 3). This stain identified four phosphorylated αA-crystallin spots of similar mass. These spots comprised a relatively small proportion of the αA-crystallin on the 2-DE gels, indicating that only a small proportion of αA-crystallin is phosphorylated in the adult zebrafish lens. No phosphorylated αBa- or αBb-crystallin was found.

### Quantitation of β/γ-crystallins

Zebrafish and mammalian β-crystallins are similar in their diversity [13]. Both taxa have an acidic and basic group whose members are well conserved, although the zebrafish genome lacks an ortholog to mammalian *βA3-crystallin*. We identified five βA-crystallins and three βB-crystallins comprising 36% of zebrafish lens total protein (Figure 1A, Table 1). Many of the β-crystallins were extensively modified. βB1 and βB2, for example, were identified as 11 and 8 spots, respectively. Many of the β-crystallin modifications differ in mass, suggesting that they are truncations. Other β-crystallin modifications are of similar mass but have different isoelectric points, which may be due to phosphorylation or other modifications. The exact nature of these modifications still needs to be examined. βB3 is the largest crystallin in the zebrafish lens due to the COOH-terminal proline-asparagine (PNPN) and proline-alanine (PAPA) repeats that are similar to the repeating PAPA sequence found in mammalian and chicken βB1-crystallin [33]. The zebrafish βB-crystallin genes are not found on the same chromosome as are the mammalian orthologs (Table 1).

Unlike the β crystallins, zebrafish and mammalian γ-crystallins have undergone greater evolutionary divergence [13]. Most noticeably, the γA-F group does not occur in the zebrafish but is replaced by at least 11 γM proteins (Table 1). Zebrafish also have four γS proteins instead of the one found in mammals. We identified protein resulting from all the γ-crystallin genes described by Wistow et al. [13] except γM5 and βγx (Figure 1B,C; Table 1), totaling 47.2% of total protein (Figure 2). In addition, we identified γM2D1- and γM2D2-crystallins. The γM-crystallins were the most abundant family in the zebrafish lens (30.5%; Figure 2). These were also the most basic γ-crystallins and the most difficult to resolve similar to the γA-F crystallins in mammals [20]. Two large unresolved γM-crystallin areas appeared when focused on the pH 3–10 nonlinear IPG strips (Figure 1B). Improved resolution on pH 7–10 strips showed that these areas also contained minor amounts of γS3-crystallin (Figure 1C). Therefore, our calculations of γM-crystallin content using the pH 3–10 nonlinear strips may slightly overestimate the total γM-crystallin content of the zebrafish lens.

### Zebrafish crystallin truncation is correlated with insolubility

**Figure 4:**
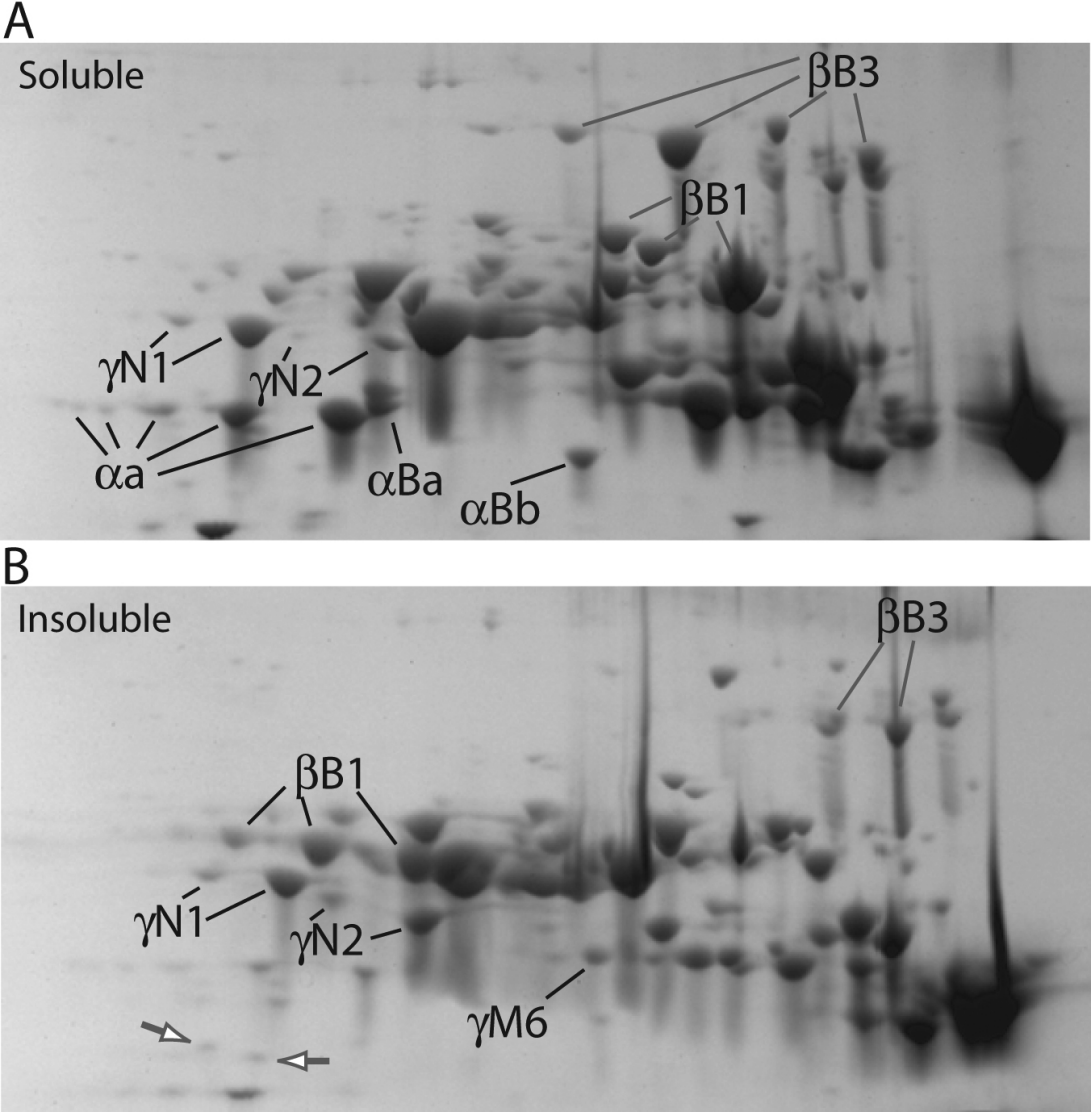
Comparison of soluble and insoluble protein from adult zebrafish lens. Both soluble (**A**) and insoluble (**B**) fractions were focused on pH 5–8 IPG strips before SDS–PAGE. Labels indicate examples of spots that are more abundant in one specific protein fraction (α-, βB1-, βB3-, and γN2-crystallin) or are equal in abundance in both fractions (γN1-crystallin). The white arrows on gel B show two α-crystallins that are preferentially found in the insoluble fraction. Many of the preferentially insoluble spots appear to be truncations.

The accumulation of protein modifications with age can decrease the solubility of crystallins and contribute to cataracts [20,24]. To determine whether the zebrafish lens will make a good model for studying the impact of crystallin insolubility, we separately quantified the proportion of zebrafish crystallins in the soluble and insoluble fractions of the lens. We found that putatively truncated proteins were more abundant in the insoluble fraction. For example, the only α-crystallin spots that were found at equal or greater abundance in the insoluble fraction were lower mass αA- and αBa-crystallins (Figure 4, white arrows). These spots were not phosphorylated, further suggesting that their insolubility is linked to truncation. All other αA-crystallin spots are much more abundant in the soluble fraction. The same pattern is found in βB1- and βB3-crystallins. Lower mass spots of these two crystallins are also found preferentially in the insoluble fraction (Figure 4). Unlike the intact α-and β-crystallins, the two γN-crystallins were not found preferentially in the soluble fraction. γN1-crystallin occurred in both fractions in roughly equal amounts while γN2-crystallin occurred primarily in the insoluble fraction (Figure 4).

## Discussion

This is the first study to quantify the proportions of individual crystallins in a fish lens and investigate their posttranslational modifications. While there are some significant differences between the crystallin content of the zebrafish and mammalian lens, most notably the lower amounts of α-crystallin and the higher amounts and greater diversity of γ-crystallins, there are also some significant similarities. In particular, similarities in α-crystallin ratios and modifications as well as the connection between crystallin truncation and insolubility support the zebrafish as a useful model for lens function and disease. This proteome map will also be of value to future studies of lens development.

Our calculation of α-crystallin content is the first accurate measurement for the fish lens. The 2-DE approach used here resolves a previously noted difficulty in separating fish α-and β-crystallins by size exclusion chromatography, which made it difficult to quantify their expression [14,17,18]. Two previous studies found α-crystallin proportions of fish lens to be 12% and 41.6% for two different species, both higher than the 7.8% reported here for the zebrafish [27,28]. It is possible that α-crystallin values do differ greatly between fish species. Future studies will need to determine if the lower proportion in the zebrafish is typical among fish species. The low proportion of α-crystallin in the rat lens (20%) compared to other mammals has been hypothesized to be due to this species’ shorter life span and subsequent reduced need for protection against age-induced protein denaturation [19]. The zebrafish has a similar lifespan of about three years. Examination of longer-lived fish species could test a possible link between α-crystallin content and lifespan. An alternative hypothesis is that the small percentage of α-crystallin in the zebrafish and rodent lenses is a reflection of their high total protein content [29]. Rodents have rigid, dense lenses that may require an increased proportion of γ-crystallin and reduced α-crystallin levels. If α-crystallin content reflects total protein concentrations, long-lived fishes should have low α-crystallin proportions similar to the zebrafish.

The large amount of γ-crystallin (47.2%) found in the zebrafish lens is similar to amounts measured in other fish species from evolutionarily diverse groups and appears to be a general feature of the fish lens [17,18]. It has been suggested that an abundance of small, polydisperse γ-crystallins allows tighter protein packing to produce the dense lenses needed for underwater vision [13,17,34]. Therefore, the expansion of the γM-crystallin family in fishes may be an adaptation for underwater vision. Our finding that γM-crystallins are the most abundant of the γ-crystallins in the zebrafish lens supports this hypothesis. Both the diversity and high concentration of γM-crystallins suggest that they are important for producing an aquatic lens with a high refractive index. In a similar way, the dense rodent lens expresses all six mammalian γA-F proteins while the softer, less dense human lens only expresses two [29] and the soft avian lens expresses none [35]. Kiss et al. [36] argue that the large number of γM-crystallins also facilitated the evolution of cold tolerance in the lens of the Antarctic toothfish, *Dissostichus mawsoni*.

Recent efforts to complete the annotation of the zebrafish genome allow the comparison of crystallin gene chromosomal locations and linkages with other vertebrate taxa. Unlike in mammals and birds, the zebrafish *βA4-crystallin* gene and the three *βB-crystallin* genes are not closely linked on the same chromosome (Table 1). The linkage of these genes in mammals and birds may be due to an original clustering of the entire β-crystallin family on one chromosome [37] as is currently seen in the mammalian γA-F-crystallins and most of the zebrafish γM-crystallins (Table 1). The dispersal of the zebrafish β-crystallins to separate chromosomes may simply reflect the evolutionary distance from the last common ancestor of teleost fishes, birds, and mammals. The possibility that chromosomal linkage between chicken *βA4-* and *βB1-crystallin* results in a mechanistic linkage in their expression [37] could mean that the expression of these genes is controlled differently in the zebrafish than in other vertebrate taxa.

Our study agrees with that of Vihtelic et al. [38], which used cDNA transcript abundance to infer that β- and γ-crystallins were the most highly expressed proteins in the zebrafish lens. However, our data also differ from this study in several ways. First, the three most abundant lens cDNA transcripts from the Vihtelic et al. [38] study were the γS1-, βB3-, and βB2-crystallins. The three most abundant crystallins in our 2-DE gels were βA1-, βB1-, and γS2-crystallin. However, since some of the 2-DE spots in our study contained several proteins, we may be underestimating the total abundance of some crystallins (Table 1). Second, this previous study found a greater abundance of transcripts for each of the two αB-crystallins than for αA-crystallin while our data indicate that αA-crystallin is the most abundant α-crystallin. And third, Vihtelic et al. [38] noted a large number of transcripts for cystatin B and suggested the possibility that this protein has been recruited as a taxon-specific crystallin in the zebrafish lens. We did not identify this protein in our 2-DE gels, although we did identify four other highly expressed non-crystallins (Figure 1), three of which were identified as cDNA transcripts by Vihtelic et al. [38] (actin, BTB domain containing protein 2, and galectin-related inter-fiber protein).

Several α-crystallin characteristics appear to be conserved between the zebrafish and mammalian lens. We previously showed that zebrafish αA-crystallin is a stronger chaperone and has greater thermal stability than αBa-crystallin, consistent with the pattern seen in mammals [39]. In this study, we show that even though the zebrafish lens expresses a third α-crystallin (αBb-crystallin) [16], the ratio of αA-crystallin to the combined αB-crystallins is very close to that of the human lens. These combined data support the conclusion that αA- and αB-crystallin play similar roles in the zebrafish and mammalian lens. The higher abundance and greater thermal stability of αA-crystallin compared to αB-crystallin in both the zebrafish and mammals reflect its similar role in preventing protein aggregation over the life of the lens. Furthermore, zebrafish and mammalian αA-crystallin share a similar phosphorylation pattern, although the exact nature of zebrafish phosphorylation still needs to be determined.

Determining the function of αB-crystallin in the mammalian lens has been problematic because it is also expressed in numerous non-lenticular tissues. Therefore, any functional analysis of mammalian αB-crystallin might not be relevant to the lens but may instead reflect function in another tissue. The presence of two αB-crystallins in the zebrafish, one lens specific (αBa-crystallin) and one ubiquitous (αBb-crystallin), provides an excellent model for discriminating between lenticular and extra lenticular functions of αB-crystallin [16]. Gene duplications are common in zebrafish due to a genome-wide duplication event early in teleost fish evolution [40]. In many cases, the functions of a single mammalian protein become divided between the two zebrafish copies [41-43]. The higher expression of the lens specific αBa-crystallin compared to the ubiquitous αBb-crystallin (2.56% and 0.72%, respectively) suggests that αBa-crystallin plays a more prominent role in the zebrafish lens. The very low chaperone-like activity of zebrafish αBa-crystallin [39] may indicate that protective function is not a primary role for αB-crystallins in the zebrafish lens. The lower expression of the ubiquitous αBb-crystallin, which is a strong chaperone [16], further suggests that the αB-crystallin protective function is of greater importance outside of the lens. If the function of mammalian αB-crystallin had been subdivided between the two zebrafish proteins, our data support the hypothesis that chaperone activity is not a prominent role of αB-crystallin in the mammalian lens.

Crystallin truncation occurs with aging in the mammalian lens and contributes to cataract [44-46]. The link between insolubility and truncation in several zebrafish crystallins suggests that this species may make a good model for studying the effects of crystallin aging on lens function. It is possible that the loss of solubility seen in our data are not directly due to truncation but instead to other modifications such as deamidation or methylation [24]. Truncation of some β-crystallins, for example, may be an adaptive response by the lens to limit the increasing insolubility caused by other modifications [47]. The preferential expression of zebrafish γN-crystallins in the insoluble fraction may be due to membrane interactions as has been shown for mammalian γE- and γF-crystallin [48]. These data could also result from γN-crystallin interaction with the cytoskeleton. Alternatively, reduced γN-crystallin solubility could be a feature of the protein itself as purified recombinant mouse γN-crystallin has reduced solubility and lower conformational stability compared to other vertebrate γ-crystallins [13]. Future studies will need to detail the β- and γ-crystallin modifications that lead to insolubility.

This study identifies several similarities between zebrafish and mammalian crystallins that support the use of the zebrafish as a model for investigations of vertebrate lens development, function, and disease. Not only can this species be used similarly to traditional models like rodents to study the adult lens, but the zebrafish also provides the added benefit of having an externally laid egg and transparent embryo, allowing in vivo observation of lens and eye development. Furthermore, a relatively low expense and ease of maintenance adds to the utility of the zebrafish. Future studies will need to further detail the posttranslational modification of zebrafish crystallins and examine developmental changes in zebrafish lens protein expression. The proteome map provided here contributes a solid foundation for these investigations.
